# Understanding the Risk of Social Vulnerability for the Chinese Diaspora during the COVID-19 Pandemic: A Model Driving Risk Perception and Threat Appraisal of Risk Communication—A Qualitative Study

**DOI:** 10.3390/ijerph21040512

**Published:** 2024-04-21

**Authors:** Doris Yuet Lan Leung, Hilary Hwu, Shoilee Khan, Aaida Mamuji, Jack Rozdilsky, Terri Chu, Charlotte Lee

**Affiliations:** 1 School of Nursing, The Hong Kong Polytechnic University, Hong Kong SAR, China; 2 Daphne Cockwell School of Nursing, Toronto Metropolitan University, Toronto, ON M5B 2K3, Canada; hhwu@torontomu.ca (H.H.); lee.charlotte@torontomu.ca (C.L.); 3 Faculty of Liberal Arts & Professional Studies, York University, Toronto, ON M3J 1P3, Canada; shoilee.khan@gmail.com (S.K.); amamuji@yorku.ca (A.M.); rozdilsk@yorku.ca (J.R.); terrichu@yorku.ca (T.C.)

**Keywords:** cognitive dissonance, COVID-19, immigrants, racism, risk communication, risk perception, qualitative

## Abstract

During the first wave of the COVID-19 pandemic, immigrants were among the most socially vulnerable in Western countries. The Chinese diaspora in Canada were one such group due to the widespread cultural stigma surrounding their purported greater susceptibility to transmit and become infected by COVID-19. This paper aims to understand the social vulnerability of the Chinese diaspora in the Greater Toronto Area, Canada, during the first wave of COVID-19 from an explanation of their risk perception and threat appraisal of risk communication. We conducted secondary data analysis of 36 interviews using critical realism. The participants self-identified as being of Chinese descent. The results were used to develop a model of how social vulnerability occurred. In brief, cognitive dissonance was discovered to generate conflicts of one’s cultural identity, shaped by social structures of (i) stigma of contagion, (ii) ethnic stigma, and (iii) public sentiment, and mediated by participants’ threat appraisal and (iv) self-reliance. We assert that risk communicators need to consider their audiences’ diverse socialization in crafting messages to modify behaviors, create a sense of responsibility, and mitigate public health threats. A lack of awareness of one’s cognitive dissonance driven by cultural vulnerability may heighten their social vulnerability and prevent them from taking action to protect themself from high-risk events.

## 1. Introduction

In North America, the initial impact of the novel COVID-19 pandemic in 2020 stretched healthcare systems, including healthcare workers, beyond their capacity [1]. National governments mandated that all citizens and residents were responsible for curbing the virus’ spread by ‘flattening the [epidemic] curve’ [2]. Indeed, prior to the COVID-19 vaccines being available (December 2019 to mid-December 2020), the only tools available to flatten the curve were non-pharmaceutical public health and social measures [3]. Examples of social countermeasures implemented in response to the spread of COVID-19 included physical distancing, travel restrictions, lockdowns, contact tracing efforts, and numerous forms of communication intended to influence behavior change [3]. Risk communication served as the foundation for rapidly putting public health measures into place to combat the pandemic threat. However, research has since reported that risk communication during that time did not address social determinants, which caused individuals to exhibit unreliable and unhealthy behaviors and impacted the effectiveness of COVID-19 control measures [4]. Social determinants of health, such as immigrant status and racism, generate a risk profile that refers to one’s social vulnerability [5]. Hence, the authors chose to explore the Chinese diaspora, a group defined as those who self-identify ethnically as Chinese through birth or ancestry, and lives outside their country of origin [6]. Moreover, the Chinese diaspora exemplified social vulnerability that impacted their health and well-being during the first wave of COVID-19 in Canada [7].

### 1.1. Context of Chinese Diaspora in the Greater Toronto Area (GTA), Ontario, Canada

The GTA is home to a diverse Chinese community who are distinguished in part by region of birth, including Mainland China, Hong Kong, and Taiwan. Mainland Chinese populations have come to outnumber those from Hong Kong and Taiwan in the last decade [8]. Chinese groups also differ according to spoken dialect (e.g., Cantonese and Mandarin), culture (including intra-ethnic stereotypes), and transnational politics [7]. Hence, this group was chosen for its heterogeneity as an immigrant group and for the multiple social barriers to healthcare that were encountered during the COVID-19 pandemic, such as English-language proficiency [7].

The GTA has a population of approximately 6.2 million [9] and includes the city of Toronto (2.7 million) as well as 10 other major surrounding cities, of which the most populated are Mississauga, Brampton, Markham, Vaughan, and Richmond Hill. The three most common ethnicities are English (12.9%), Chinese (12.0%), and Canadian (11.3%), of whom just under half are non-native migrants (i.e., settlers or immigrants) [9]. The Chinese population in the GTA is one of the world’s largest, estimated at 679,725 as of the 2021 census, second only to that of New York City in the USA [9].

As the first wave of the virus spread amongst the community in Toronto during the winter and spring of 2020, with COVID-19 beginning to surge in March 2020, the occupancy rate of acute care hospital beds in the GTA reached 93% [10]. Certain residents were at greater risk of COVID-19 (1.3 to 1.8 times higher than the white community) as a result of their living and working conditions, including lower income, identification with a racialized group, and living in crowded housing [9]; one such racialized group was the Chinese diaspora, who were more likely to report mental health-related phenomena as compared to their white counterparts in Canada [7].

### 1.2. Chinese Diaspora in Canada during the COVID-19 Pandemic (2020)

Immigrants from over 450 ethnic and cultural backgrounds have made Canada their home [10]. In a census taken in 2021, visible minorities account for 16.1% of Canada’s total population, of which 4.7% (1.7 million) self-identify as Chinese [11]. During the first wave of COVID-19 in Canada, certain groups within the Chinese diaspora reported increased mental health problems relative to their dominant white counterparts [12]. Specifically, a survey of 471 immigrants from Mainland China conducted from April to June 2020 reported that 11.3% experienced depression and 10.8% experienced moderate to severe anxiety associated with worry about contagion for oneself and one’s family ‘all the time or often’, concerns or confusion about the authenticity of Canadian measures, information confusion, and contagion prevention measures (i.e., food/goods stocking, and room cleaning/sanitizing) [12]. These results suggest that Chinese immigrants’ mental health vulnerability was located within their work of navigating intergroup relations and information of cultural/national conflicts [7]. Moreover, Gao [7] argued that identity and belonging appear related to international political tensions, such that COVID-19 scapegoating lay blame to those of Chinese descent for carrying and spreading the virus [7]. This generated a sense of social vulnerability from public stigma (a social structure) created by negative labels to ‘a shared ideological recognition of national/cultural identity’ [7] (p. 214). Indeed, groups identifying as Chinese migrants (including settlers with a heritage of one or more generations) experienced concern for overt cultural racism during the COVID-19 pandemic [7], which was likely detrimental to their mental health [12].

Cultural racism is a form of structural racism that Dean and Thorpe [13] define as ‘reflecting the ideologies and societal norms about a particular racial/ethnic group, which situates them to be more vulnerable and shape their trust of the broader public, due to historically rooted and culturally reinforced inequitable systems’ (p. 1523). Canada’s Chinese migrants were intimately connected—culturally, socially, and politically—to China, where the virus originated [12]. These provoked fears of racism motivated not only by symbolic perceptions of them as a potential source of infection but also as being responsible for the pandemic [12]. As such, Canada’s Chinese communities reported experiencing cultural racism that was almost fivefold (25%) that experienced by the rest of the Canadian population (6%) [14]. Additionally, 11.3% of Mainland Chinese (more often younger, female, and of lower education) in Canada reported moderate to severe levels of depression, while 10.8% reported severe levels of anxiety during the first wave (April to June 2020) of COVID-19 [12]. Indeed, cultural racism shaped Chinese persons’ perception of risk communication [7,12].

### 1.3. Risk Communication and Risk Perception

Risk communication is a sustained communication process intended to engage and persuade a diverse audience by raising awareness of health threats and equipping them to make informed decisions and protect themselves against infection, that is, to engage in threat appraisal [2,15]. During the first wave of COVID-19, risk communicators stated, ‘if disease precautions are adhered to, the result would theoretically spread out [or ‘flatten’] how many people would get sick over time, so that hospitals and other resources would not be exhausted’ [16] (p. 1). Risk communication assumes that authorities will provide credible information based on individuals’ risk perception and assist access to needed resources in consideration of individuals’ social contexts.

Risk perception is the awareness of and receptivity to one’s vulnerability to threat and the need to appraise, mitigate, and cope with potential harms [4]. Risk perception is shaped by individual beliefs, which may (or may not) align with state ideologies; thus, value conflicts between the self and the state can generate a sense of incapacity (e.g., helplessness, resistance, and rebellion) to control risks [17]. Indeed, according to Gesser-Edelsburg [18], fear-based strategies to amplify the risk of morbidity and mortality may motivate the public to resist public health guidelines, as they may not trust the information given by risk communicators who do not offer solutions that accommodate groups that experience health disparities due to social inequities [18]. Hence, individual risk perception is shaped by one’s awareness of and receptivity to the question of whether one’s vulnerability can be prevented or controlled, dependent not only on one’s agency [self-efficacy] but also on how it intersects with the social determinants that shape our attitudes and beliefs [response efficacy] [18,19,20].

Based on these components, Maloney, Lapinsky, and Witte [20] suggest that two components constitute one’s threat appraisal: the perceived severity (i.e., magnitude) and susceptibility to encounter the threat (i.e., called the Extended Parallel Process Model). Variability in one’s risk perceptions was never more evident than during the first wave of COVID-19, when healthcare policies (e.g., no sick leave) mediated how illness (and risk) was differentially perceived and responded to, depending on one’s situation (e.g., frontline healthcare workers versus those enabled to work from home) [21].

Wittenberg et al.’s [22] systematic review of COVID-19-related communication between healthcare providers and patients (1 January 2020–1 September 2020) identified a dearth of culturally sensitive public health communication for informal caregivers, such as family members. Sutton et al. [17] similarly asserted that ‘without recognizing social determinants that facilitate individuals’ lived realities, public health messaging is likely to be ineffective and could potentially exacerbate existing health disparities’ (p. 373). Our study targets the intersection between individual risk perception and social determinants (e.g., communication infrastructure), together which influence the attitudes and beliefs governing conformity to risk communication.

### 1.4. Research Gap and Study Purpose

Risk perception research to date has largely failed to consider how one’s social and economic positions, education, life experiences, and skills intersect to trace a trajectory toward social vulnerability—‘placing one more or less subject to exclusion processes’ [23] (p. 28). According to Mah et al. [24], social vulnerability is ‘defined as the degree to which a person’s overall social circumstances (e.g., poverty, isolation) leave them unable to respond in a crisis, and therefore, susceptible to insults (i.e., health or socially related adverse events)’ (p. 447). Indeed, one’s social vulnerability is constructed *with* others and lies within perceptions of risk in their environment—in this case, COVID-19 contagion—and their efficacy (confidence of one’s own capacity and/or with others) in terms of responding with precautionary behaviors [25]. Risk communication thus requires approaches that go beyond individual-focused, fear-based messaging to establish new normative, shared values and attitudes aimed at helping communities become resilient in the face of prolonged health emergencies [7,18].

Adekunle and Mohammed [26] emphasize the importance of contextualizing health risk communication in relation to the cultural intersections inherent in social structures (such as stratification of social class) and individual agency (i.e., a temporal capacity of one’s own social and local circumstances). The ways in which certain groups trust, maintain social cohesion, generate community participation, and assign ownership for self-protective behaviors can inform future pandemic response efforts [1]. Hence, our aim was to understand the social vulnerability of the Chinese diaspora in the Greater Toronto Area, Canada, during the first wave of COVID-19 from an explanation of their risk perception and threat appraisal of risk communication. In doing so, we sought to develop a theoretical explanation of such vulnerability and to enhance risk communication during public health emergencies, which may be transferable to similar socially vulnerable groups.

## 2. Materials and Methods

### 2.1. Study Design and Research Question

This study used a qualitative design. We used data from a previous qualitative descriptive study exploring emergency management actions to address anti-Chinese stigma and the social vulnerability of the Chinese diaspora in the GTA, Canada, during the first wave of COVID-19 [27]. By conducting secondary data analysis, we were able to pursue a different research question: What mechanisms and structures underpin risk perception and threat appraisal associated with pandemic challenges for diasporic Chinese in the GTA, Canada?

### 2.2. Theoretical–Conceptual Framework

Unlike previous research, we approached the study using a critical realist paradigm. Critical realism is a scientific philosophy developed by Bhaskar in the 1970s [28]. Unlike other qualitative paradigms, critical realism acknowledges the value of both experimental and interpretivist lenses through ontological assumptions (meanings of ‘being’) on the grounds that realist and epistemological assumptions (i.e., how knowledge comes to be known) are relativist [29]. This paradigm is used for studies of social sciences and social psychology [30] and health [31]. We selected this paradigm for its fit to a causal explanation of a phenomenon, one concerned with deeper mechanisms and wider determinants that underpin individual agentic capacities in interaction with their contextual circumstances [32].

Critical realism seeks to uncover how, why, for whom, and under what circumstances mechanisms generate outcomes within three domains of reality [28] stratified into social layers (strata) called the ‘empirical, the actual, and the real’. According to Elder-Vass [28], the first and most evidential is the domain of the ‘empirical’, which concerns our experiences of feeling, thinking, observing, and talking about events. The ‘actual’ domain consists of the tangible or material interactions or chain of events that we may or may not be conscious of happening [28]. Finally, the ‘real’ consists of an ensemble of causal mechanisms and powers in our relationships (social entities), all of which are revealed in how we make sense of our events and/or experiences—that is, at the level of the ‘empirical’ [28].

### 2.3. Researcher Characteristics and Reflexivity

Six of the seven research members identified as belonging to a visible minority (DL, CL, HH, TC, SK, and AM) and four (DL, CL, HH, and TC) identified as Chinese immigrants from first to fourth generation in Canada. Two academics (AM and JR) were experienced in disaster and emergency management research. Altogether, our professional and personal experiences were a resource for research analysis, though care was taken not to let our experiences dominate participants’ narratives. (Please see techniques to enhance trustworthiness).

### 2.4. Recruitment of Participants

The recruitment of participants during the first wave of COVID-19 (March–May 2020) occurred through professional and social networks. Specifically, outreach strategies were used to recruit participants, including postings on the project’s website, using the research team’s social media accounts, and the use of snowball sampling. Eligible participants responded to the researchers’ contact emails or through their phone numbers. The inclusion criteria were (i) self-identifying adult Chinese immigrants; (ii) residency in the GTA; and (iii) willingness to participate in an interview in their preferred language. Beyond this, no exclusion criteria were applied. As researchers of the primary study [27] anticipated a diverse group of immigrants based on multiple social determinants of age, sex, education, employment status, language, country of origin, citizenship status, neighborhood, and experience of discrimination, a larger sample size of at least 80 participants was initially targeted (10 participants in each group) for data sufficiency. However, in this secondary data analysis, theoretical saturation became the focus; thus, we estimated that 35 of the 83 interviews previously collected would achieve conceptual adequacy [33]. We purposefully selected the participant interviews following maximum variation in intersecting social determinants: age, self-identifying gender, country of origin, occupation, education, years residing in the GTA, and those reporting direct or indirect experiences of racial discrimination during the first wave of COVID-19 (i.e., 30 of the 83 participants). Informed consent procedures were followed and documented by researchers prior to data collection.

### 2.5. Data Collection

The data from the original 83 interviews were collected by phone, Zoom, Google Meet, or Skype (audio only) in English (64 interviews), Mandarin (5 interviews), or Cantonese (14 interviews). Interviewers used a short questionnaire to collect socio-demographic information, followed by an interview using a semi-structured guide developed by two researchers (AM and CL). The questions drew on pre-existing constructs in the literature (i.e., risk communication, risk perception, social determinants, cultural racism, and stigma) while remaining open to the participants’ reported challenges associated with COVID-19. Specifically, open-ended questions were guided by the following queries:

- How is COVID-19 impacting you and your family’s everyday life?
- Where do you seek information about COVID-19?
- How do you feel about the COVID-19 information that you receive?
- What are the challenges of COVID-19 that you or your family/friends face?

Prompts were used to gather more information following each participant’s lead, exploring the credibility of the information and the resources used to manage challenges. All interviews were audio-recorded and transcribed verbatim. Transcripts were then translated (and verified) into English for analysis by two researchers fluent in both Chinese and English. It should be noted that although most (83%) of the interviews were conducted in English, 17% (n = 6/36) required translation prior to analysis as the lead author/researcher was not fluent in Chinese. NVivo12 software supported the data management and organization [34]. Theoretical saturation was achieved after 36 interviews, verified by three research members (DL, HH, and SK) when no new theoretical patterns occurred after the 34th interview. Interviews lasted between 30 and 60 min (average of 45 min) [33].

### 2.6. Data Analysis

Data analysis was conducted by three researchers (DL, HH, and SK) who moved back and forth between four levels of thematic analysis in accordance with Fryer [35]: (i) familiarization with the data; (ii) applying, developing, and reviewing codes; (iii) developing and reviewing themes; and (iv) generating conclusion and reports. The lead author led familiarization of the data with at least one other researcher, skimming all data and documenting initial thoughts, questions, associations, and surprising elements in a memo document.

The second level of analysis assigned codes to chunks of text. Initial coding was applied to 10 percent of the original 83 interviews (9 of them) to develop preliminary descriptive constructs [35]. Each researcher coded interviews independently and then compared the codes to reach consensus by at least two researchers (the layer of the experiential or ‘empirical’). These codes informed the initial descriptive structure of participants’ experiences that we would build on in subsequent data-led reviews.

The third level of analysis constituted an iterative process of cycling back and forth between familiarization, developing codes, and developing themes. This involved grouping similar codes together in what Fryer [35] calls ‘standardization’ of codes. ‘Consolidation’ entailed thinking about what theoretical or general terms might be used in descriptive codes to address the research question at the level of the ‘actual’ [35].

The fourth level generated conclusions and reports. This required reflection on the overall analysis for trustworthiness (see below) and the theoretical validity of conclusions. The researchers used retrodictive (deductive) reasoning of theoretical constructs from previous research to consider the explanatory power of themes posited to generate material or (in)tangible events (layer of ‘the actual’), forming multiple mechanisms and structures (layer of ‘the real’) [35].

### 2.7. Trustworthiness

Trustworthiness was cultivated through several techniques to establish four criteria, according to Fryer [35]: (i) credibility; (ii) plausibility; (iii) transferability; and (iv) utility or impact. Credibility and plausibility were assured through independent and comparative coding of the first ten percent of transcripts to create a descriptive coding structure for the rest of the analysis. Thereafter, each new code generated from the next set of transcripts was confirmed by another researcher. Plausibility of theorization occurred through active dialogue and reflection between research members, and with extensive knowledge of qualitative research and the related literature. Finally, transferability is assessed by readers of this paper, who may compare findings against the wider literature and their own contexts. Utility or impact was assessed through analysis of how findings agreed or disagreed with extended knowledge of the phenomena, thereby reconceptualizing the main theoretical construct. O’Brien et al.’s [36] EQUATOR: Standards of Reporting Qualitative Research (SRQR) was followed to ensure transparency and comprehensiveness.

### 2.8. Ethical Aspects

Prior to data collection, ethical approval was granted by the respective research ethics boards of the participating universities, and verbal, informed consent was obtained from all participants. Confidentiality was ensured by removing identifiers (i.e., names and places) prior to analysis. All data were stored in password-protected computers and secure internet servers. Participants were identified exclusively by assigned numbers.

## 3. Results

### 3.1. Participants’ Socio-Demographic Characteristics

Thirty-six participants participated in this study. Their characteristics are summarized as follows: 64 percent of participants were aged between 25 and 64 years; 42 percent had immigrated from Hong Kong, 31 percent from Mainland China, 8 percent from Taiwan, 5 percent from other Asian countries, and 14 percent did not specify (including those five percent born in Canada); 53 percent were employed; 64 percent identified as female; and 75 percent had lived in the GTA for six years or more. Seventy percent reported having experienced direct or indirect stigma, racism, or microaggression during the COVID-19 pandemic. Please see Table 1 for further details (below).

### 3.2. Thematic Results: Summary

**Figure 1:**
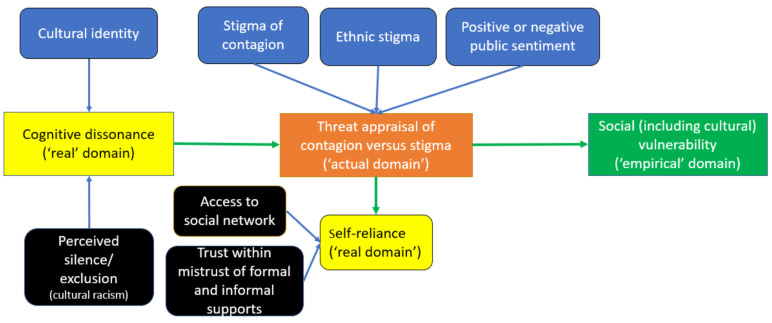
Theoretical model of Chinese diaspora persons’ risk perception and threat appraisal generating their social vulnerability during the first wave of COVID-19.

The theoretical model developed allows for a description to emerge to shed light on the risk perception and threat appraisal situation present. (Please see Figure 1 below). The model first characterizes social vulnerability, which includes cultural vulnerability. Second, those vulnerabilities arise from threat appraisal of contagion versus stigma. Third, the model then allows for a description of the key driving mechanism theorized at the onset of this phenomenon, which is cognitive dissonance.

### 3.3. Social Vulnerability

All participants reported some degree of perceived social vulnerability in their environment. That social vulnerability was influenced by direct or indirect insults regarding comments on the COVID-19 situation reflecting attitudes of anti-Chinese sentiments or Sinophobia. Historical and political biases of their assumed cultural identity shaped this vulnerability, such as when a recently immigrated woman from Hong Kong, encountered a neighbor who stated the following:

*‘So, we’re wearing masks, and a French lady neighbour came up to me and warned me not to wear masks without even asking me whether someone was sick. Just told me that “Oh you better not wear a mask to avoid the stigma because you’re Asian and wearing a mask.” I thanked her for the concern.’*

(#24)

Participants were thus stereotyped with an implicit cultural identity tied to their Chinese heritage that differentiated them from mainstream (white) Canadians. Indeed, participants were reminded that their mere presence posed a potential threat, not only of COVID-19 contagion but also of public stigma, associated with an (im)moral responsibility for having caused the pandemic based on their ethnicity.

### 3.4. Individuals’ Threat Appraisal of Stigma Versus Contagion

Participants’ threat appraisal comprised perceptions of consequences of contagion compared to consequences of ethnic stigma. This emergent mechanism consisted of four parts: (i) stigma of contagion, (ii) ethnic stigma, and (iii) public sentiment, altogether mediating a mechanism of (iv) self-reliance toward their social sphere (including cultural vulnerability). Participants’ self-reliance was shaped by their access to a social network and their trust (within mistrust) of their formal and informal supports. All participants reported that their appraisal of contagion versus stigma varied between mild and extreme.

(a) Stigma of contagion

The threat of contagion was dynamic, subject to rapidly changing information. Participants reported having sought and appraised information that influenced whether they adopted self-protective behaviors, including wearing masks in public. One student from Mainland China [resident in the GTA for 9 years] stated that

*‘I was saying from my observation a lot of, like most Chinese people, like vast majority of Chinese people, wear masks. But like […] Canadians, a lot of them still don’t really wear masks.’*

(#40)

For all participants, the threat of contagion was appraised based on contexts (e.g., at work, home, and public transit) temporal to how they were situated and how behaviors (e.g., masks) made sense to them.

(b) Ethnic stigma

Initially, the issue of COVID-19 contagion appeared to dominate the threat of stigma, as the negative consequences of contracting the virus provoked fears of self-blame and public shame. As one older participant [who immigrated to the GTA as a young child with his family] stated,

*‘So, if you have one member of your family that doesn’t follow the rules, he is the killer. He is the one that caused problems because he’s the weakest guy. He’s the guy who is most likely to catch the virus and, in turn, will give it to the rest of the family.’*

(#12)

By contrast, some participants reported worrying about a prolonged threat of stigma, rooted in anticipated political and internalized biases of the public toward themselves as a Chinese person. These biases were cumulative from past pandemic experiences: one participant [resident in the GTA for 30 years] compared the current pandemic to past episodes of cultural racism during the 2009 H1N1 pandemic:

*‘I remember in 2009, there was H1N1, it originated in Mexico; they were the ones who got it first. Then it spread to many countries. I paid attention to the news at that time to see if anyone humiliated Mexican. I found out that there was none. And when it comes to COVID-19, many racists scolded, humiliated, and even hit Chinese. This is not fair for Chinese.’*

(#38)

The degree to which participants appraised the potential threat of contagion versus stigma appeared to depend, in part, on current public sentiment.

(c) Public sentiment

Participants’ assumptions about their vulnerability depended on societal beliefs about socio-economic class and were shaped by authorities’ language and tone and/or reports of public sentiment in Canadian social media compared to elsewhere, in particular, the negative anti-Chinese sentiments related to COVID-19 in the USA. As one well-established professional [living in the GTA for 36 years] stated,

*‘It really seems to me it’s about the leadership of where one is. And when I think about Trump it’s not just that he normalizes this casual racism … But I also think about Chinese people under him just getting to be more afraid even if nothing bad actually happened because they feel that target and they feel not supported in that way and I hope that people here feel a contrast, that the Chinese community feels differently.’*

(#25)

Participants’ appraisal of threat and, thus, their sense of vulnerability was mediated by their self-reliance and the quality of their social support. Participants’ social networks could strengthen or weaken their self-reliance (i.e., both a personal and cultural disposition) and thus their resilience when encountering threats.

(d) Mechanism of self-reliance to recover from adversity

Participants had different types of social networks with varying degrees of accessibility. As such, the capacity and degree to which the mechanism of self-reliance mediated their social vulnerability depended on two factors: (i) their access to a quality (strong) social network, rather than the size of that network; (ii) trust within mistrust of formal and informal supports.

Access to social networks. Individuals with less access to formal supports were understandably more dependent and strongly influenced by informal contacts (or lack thereof), as one participant from Mainland China [living in the GTA for 40+ years] stated,

*‘They’re [parents] not able to go out and they hear the news, and unfortunately, most of the time, the news, it’s not too positive. They don’t have a lot of friends, so they do feel anxious and nervous because they only hear the negative news, and they don’t have a lot of friends among their network to alleviate their worry.’*

(#34)

An individual’s reliance on their social network reduced their perceived threat level, as social networks functioned to allow community members to help others meet their own needs. For example, helping others with the essential need of grocery shopping during the pandemic ultimately served to reduce perceptions of threat. As one participant from Hong Kong [resident in the GTA for 22 years] stated,

*‘Like an old person says, “hey, I need a grocery run” and then someone will respond. And then once it’s resolved, the thread is closed.’*

(#14)

Trust within mistrust of formal and informal supports. Participants questioned the veracity of information from formal and informal sources (i.e., their source, credibility, and details) as much as they continuously sought out alternative evidence to make sense of it. As one participant [who recently immigrated to the GTA] stated,

*‘Because I came from Hong Kong, I experienced SARS. When it seriously contradicts my opinions, I tend not to believe it, or I would look at and check with the media that I can trust.’*

(#66)

Hence, individuals enacted self-reliance to the degree that their formal supports (e.g., physicians and nurses) and informal supports could be trusted to provide support (practical and emotional).

### 3.5. Risk Perception: Driving Mechanism of Cognitive Dissonance

A key causal mechanism was individuals’ cognitive dissonance, which moderated their social vulnerability. The degree to which a participant experienced cognitive dissonance reflected two orientations: (i) their cultural identity as belonging (as an insider or outsider) to a Chinese community and (ii) perceived silence/exclusion (cultural racism). We coded cognitive dissonance in reference to the mental discomfort/tension associated with holding two conflicting beliefs, values, or attitudes at odds with one another, driving tendencies to seek information and resources consistent with their attitudes and perceptions to ease this conflict [37].

Participants’ tension arising from cognitive dissonance was revealed in their social comparisons with other countries, ethnicities, family, and friends in their efforts to make sense of information and ease conflict about their social vulnerability. Comparisons appeared to either reinforce their perceptions of themselves as discriminated against, as undeserving of discrimination, or as ‘lucky’ to avoid discrimination. As one retired participant [residing in the GTA for about 40 years] stated,

*‘I’m quite blessed, right. I haven’t been discriminated against, even before…but I would not disregard it as unimportant. I think those who treat people badly are just ignorant. To me, COVID has no boundary. No race. Nothing, right? So, everybody can catch it.’*

(#35)

As this suggests, participants’ concerns centered around whether they were ‘lucky’ or ‘unlucky’ to have experienced cultural racism, as they acknowledged that their visible identity did not fit the wider social norms of an Anglo-Saxon ethnicity within Canadian culture.

(a) Cultural identity

Participants’ cultural identity manifested in their cultural dispositions to wear masks, perceived to be risking their physical safety as mandatory guidelines did not endorse wearing them. One self-identified activist [who immigrated to the GTA five years ago] described it in the following way:

*‘We have this debate, and I am telling him that you shouldn’t buy masks for everyone because of public relations. I don’t want people to see Chinatown purchase a bunch of masks and give it to frontline people, because I know we’ll be attacked.’*

(#7)

As this excerpt suggests, participants’ cognitive dissonance manifested in appraisals of whether the information they received was indicative of how their attitudes and values belonged (or not) to wider social norms as Canadian citizens/residents. Notably, that which was conveyed by public authorities was as important as that which was not conveyed.

(b) Perceived public silence and/or exclusion in groups (cultural racism)

The greater the participants’ cognitive dissonance, the greater the threat—and thus the vulnerability—that they perceived. This appeared tantamount to the anticipated impact on themselves and their close contacts based on their identification as Chinese, and whether the government was cognizant of their needs for protection from public stigma. As one PhD student participant from Mainland China [recently residing in the GTA] stated,

*‘It’s a feeling of not having much understanding and support around you, it’s even sadder. Recently, a white Canadian classmate told me… “Whatever you said at that time is now happening here.” She is also sad, and she is unsure why the so-called mainstream, so-called official, so-called government here in Canada had not been able to hear the voices of the Chinese and the emotions of the people like me.’*

(#80)

Indeed, participants compared their own vulnerability to that of other groups excluded from public health considerations, such as any person not fluent in English, all of whom had encountered social barriers to their health security. One young participant, who had immigrated [seven years previously] with her older parents from Mainland China, stated that

*‘People like my parents who do not speak much English only receive information from Chinese sources so they do not know much from the Canadian side, and maybe they are just hearing about the numbers and scared but they are not hearing all the other information that they do not understand in English.’*

(#42)

Prolonged cognitive dissonance appeared to generate an intolerable tension for participants for lengthy periods of time, often requiring them to take breaks or distract themselves from news of the COVID-19 pandemic. As one older self-employed participant [who had immigrated to the GTA from Hong Kong 24 years] stated,

*‘In the beginning, I was very serious… But after a while, I felt like I couldn’t bear it anymore. It was very hard. So, I read something funny. I watched some Hong Kong comedy from before, and later, I also cooked. I cooked and talked about things related to cooking and other more positive things. Tried to feel everything is okay, and I also listened to the radio.’*

(#41)

The participants reported that regulating their consumption of COVID-19 information was challenging but necessary to protect their mental health and well-being.

## 4. Discussion

The COVID-19 pandemic exacerbated Chinese immigrants’ social vulnerability. Their strength from their cognitive dissonance—originating from their cultural identity and cultural racism— moderated their continual threat (re)appraisals in their everyday lives. Our findings resonate with those of other studies concerning the first wave of COVID-19, offering evidence for the social vulnerability experienced by non-native groups, such as Latinx and Indigenous Mexican immigrant communities in the Inland Southern California desert [38] and minorities in Antwerp, Belgium [39]. However, our study demonstrated that participants’ cognitive dissonance emerged, in part, through risk communication grounded in social structures that ignored culturally held values of heritage and social equity.

Our findings extend our knowledge of how perceptions of exclusion can erode the effectiveness of risk communication, causing social vulnerability for diverse individuals. In our study, the added distinction of cultural vulnerability, as part of social vulnerability, appeared to manifest as the reverse of cultural safety. According to Curtis et al. [40], cultural safety is an ongoing and reflective process focused on critical consciousness of power in relationships and a person’s rights. We revealed cultural vulnerability to be the absence of societal or systemic reflexivity regarding how power manifests in structural relationships (embodied in cultural stereotypes of language and action) and hinders one’s rights, such as access to knowledge or practices to keep one healthy and safe. As Vargas, Mora, and Gleeson [41] reported, racialized differences beyond religion and political ideology in the USA substantially influenced the perception of the danger posed by COVID-19. However, the full extent of cultural vulnerability may be obscured because culture and its relations tend to be conflated with social capital/systems (i.e., social networks, support, and integration) rather than viewed as an evolving process rooted in our primary socialization [42].

Archer’s [43] seminal work asserted that cultural systems are ‘constituted by the corpus of existing intelligibilia—by all things capable of being grasped, deciphered, understood or known by someone’, though the tendency for ‘inclusion of components depends on this dispositional capacity alone, and not on whether contemporary social actors are willing or able to grasp, know, or understand them’ (p. 504). Our findings reinforced social vulnerability’s contingency on prior dispositions of cognitive dissonance, culturally influenced by interactions with one’s environmental and personal circumstances (both current and historical). To expand, social vulnerability was connected to relational assumptions inherent in one’s social systems—that is, social agentic capacities shaping risk perception. Our participants’ preconditions of (mis)trust and self-reliance were value-based but also culturally acquired through socialization—that is, through comparisons of fitting (or misfit) with larger social and political norms, generating their cognitive dissonance.

The term ‘cognitive dissonance’ was first coined by the social psychologist Festinger in 1957 [37]. According to Cooper et al. [37], the phenomenon has since been found to create the most tension when three components are present: choice (decision freedom), high commitment (responsibility), and anticipation that the event will yield adverse consequences, if foreseeable at the time of choice; further, the cognitive dissonance pathway is initiated if and when one contemplates actions that conflict with their social norms/values/beliefs, leading to cognitive dissonance arousal—that is, ambivalence or tension. Through social comparison to similar others, one can appraise the likelihood of an adverse consequence for which one can take responsibility for choosing to act (or not act) at that time (decision freedom). This will diminish one’s cognitive dissonance, hopefully stave off the adversity, and prepare for effective action [37]. Our study extends the conceptual clarity of how social vulnerability emerges to include cultural vulnerability when driven by cognitive dissonance [44].

### 4.1. Implications for Health Policy

According to Vaidis and Bran [44], the cognitive dissonance pathway has been underexamined and inconsistently operationalized in social psychology. Some of our study participants struggled with their cultural identity against social norms and whether to rely on information from authorities during the first wave of COVID-19. Their cognitive dissonance may have heightened their risk awareness of encountering at least two adverse consequences: contagion versus public stigma. Indeed, the extent to which cognitive dissonance generates a threat appraisal (risk perception) is critical to how individuals determine whether health and social policy reflects the best course of action in accordance with their disposition or values [45]. As Gesser-Edelsburg [18] stated, public willingness to follow risk communication is dependent on the degree that the public trusts that the state has their best interests (based on their circumstances and values) in mind.

Meanwhile, cognitive dissonance can also motivate individuals to alter their (cultural) beliefs, attitudes, and behaviors to satisfy the drive ‘to have correct and appropriate opinions’ [37] (p. 1). Thus, cognitive dissonance is caused not by inconsistency per se but by the perception of responsibility and one’s ability to act to avoid an adverse event. Hence, ‘we are not driven to reduce inconsistency per se, but rather driven to have an unambivalent stance toward the world to prepare us for effective action’ [37] (p. 4). Indeed, cognitive dissonance may be a protective mechanism felt directly or vicariously through others that may trigger us to change our attitudes and behaviors to better adapt to a risk [37]. In a second paper, we shall explain how the social vulnerability of the Chinese diaspora was impacted by participants’ different cognitive dissonance pathways.

### 4.2. Limitations

This study explored the phenomenon of risk communication during the initial wave of the COVID-19 pandemic, which was found to differ from past pandemics [18], and as such, the findings are not comparable with them. The majority of participants in this study had internet and media accessibility and sufficient astuteness to comprehend risk communication, thus limiting the findings’ generalizability to those who did not. Hence, the findings may not apply to those who required alternative strategies to access public health risk messages or were not concerned to the same degree as those in developed countries with various social media [17].

In addition, the sample size of this qualitative study is necessarily small and represents a select group, whom were for the most part under 64 years old and female. Hence, the findings may be transferable to similar groups, but are not generalizable to all people from Chinese communities nor to other ethnic minorities in Canada.

Finally, English translation was necessary for some (17%) Chinese transcripts prior to analysis, and while caution was taken to verify preliminary interpretation with researchers who were fluent in both English and the participants’ Chinese dialect, some risk remains that cultural nuances may have been lost or altered during the analysis process.

## 5. Conclusions

The conclusions of this study investigating COVID-19′s impacts on the Chinese community in the GTA, Canada, are suggested to support interpretations of cognitive dissonance, as stated by Coooper [37]. Cognitive dissonance is shaped by dispositions of cultural (and social) identity. Individuals who hesitate or avoid action as a result of their ambivalence and extreme self-reliance may not sufficiently protect themselves from adverse events unless convinced that they can successfully deflect the adversity (e.g., contagion and/or stigma) anticipated at the time of their decision to act [37]. Similarly, if an individual’s appraisal of the threat is not felt to be their responsibility (e.g., the threat of contagion is low), owing to uncertainty surrounding its very existence or the likelihood that they will encounter it, the individual will likely not commit to taking action [37]. Indeed, cognitive dissonance can prevent individuals from acting in accordance with their values [46]. Hence, in preparing for future health emergencies, risk communicators must consider the socialization of individuals.

In a study conducted in India during the COVID-19 pandemic, cognitive dissonance was theorized as a possible means of hindering social networking usage due to the imposed social norms of physical distancing [47]. In other words, one’s disposition to seek help was constrained by their inner conflict, were they to disrupt perceived social norms. As such, we assert that risk communicators need to consider their audiences’ diverse socialization in crafting messages to modify behaviors, create a sense of responsibility, and mitigate public health threats. A lack of awareness of one’s cognitive dissonance driven by cultural vulnerability may heighten their social vulnerability and prevent them from taking action to protect themself from high-risk events.

## Figures and Tables

**Table 1:** Socio-demographic characteristics of participants (n = 36).

	Frequency (n)	Percentage % (Rounded Up)
Age		
≤18 years	7	19
25–64 years old	23	64
65–74 years old	6	17
Self-Identified Gender		
Female	23	64
Male	13	36
Living in the Greater Toronto Area		
1–5 years	9	25
6+ years	27	75
Self-Identified Ethnic Origin		
Hong Kong	15	42
Mainland China	11	31
Taiwan	3	8
Other Chinese	2	5
Did not specify	5	14
Employment Status		
Employed	19	53
Unemployed	6	17
Student	5	14
Retired	6	17
Self-Reported Stigma/Racism/Microaggression during COVID-19		
Yes, direct	19	53
Yes, indirect	6	17
Denies	11	30

## Data Availability

The data are contained within the article.
